# Intergroup-statement: statement of the german ovarian cancer commission, the North-Eastern German Society of gynecological Oncology (NOGGO), AGO Austria and AGO Swiss regarding the use of homologous repair deficiency (HRD) assays in advanced ovarian cancer

**DOI:** 10.1007/s00404-025-07991-y

**Published:** 2025-03-12

**Authors:** Lukas Chinczewski, Philipp Harter, Lukas Heukamp, Doris Mayr, Christoph Grimm, Viola Heinzelmann-Schwarz, Pauline Wimberger, Sven Mahner, Ioana Elena Braicu, Wolfgang Schmitt, Carsten Denkert, Jalid Sehouli

**Affiliations:** 1 Department for Gynecology, Campus Virchow-Klinikum, Charité–Universitätsmedizin Berlin, Berlin, Germany; 2 Department for Gynecology, Klinikum Essen-Mitte, Essen, Germany; 3 Institut für Hämatopathologie Hamburg, Hamburg, Germany; 4 Department for Pathology, Ludwig-Maximilians-Universität München, Munich, Germany; 5 Department for Gynecology, Allgemeines Krankenhaus Wien, Vienna, Austria; 6 Department for Gynecology, Universitätsspital Basel, Basel, Switzerland; 7 Department for Gynecology, Universitätsklinikum Carl Gustav Carus Dresden, Dresden, Germany; 8 Department for Gynecology, Ludwig-Maximilians-Universität München, Munich, Germany; 9 Department for Pathology, Campus Charité Mitte, Charité–Universitätsmedizin Berlin, Berlin, Germany; 10 Department for Pathology, Philipps-Universität Marburg, Marburg, Germany

**Keywords:** Gynecological oncology, Ovarian cancer, Homologous recombination deficiency testing, Maintenance therapy, Intergroup statement

## Abstract

**Introduction:**

Homologous recombination deficiency (HRD) is a key biomarker in the management of high-grade serous ovarian cancer (HGSOC), guiding treatment decisions, particularly regarding the use of poly(ADP-ribose) polymerase inhibitors (PARPi). As multiple HRD assays are available, each with distinct methodologies and cutoff values, the interpretation and clinical application of HRD testing remain complex. This intergroup statement, endorsed by the German Ovarian Cancer Commission, NOGGO, AGO Austria, and AGO Swiss, aims to provide guidance on the indications, appropriate use, and limitations of HRD testing in ovarian cancer.

**Materials and methods:**

The statement is based on an interdisciplinary review of available literature, clinical trial data, and expert consensus. The recommendations focus on the current landscape of HRD assays, their clinical applicability, and practical considerations regarding the optimal timing and indications for testing.

**Results and discussion:**

Various HRD assays, including established commercial tests and emerging academic-clinical approaches, are reviewed in this statement. The document outlines key eligibility criteria for HRD testing in ovarian cancer, emphasizing its relevance in specific histological subtypes and clinical scenarios. Additionally, exclusion criteria are defined, highlighting cases where HRD testing may not be appropriate due to insufficient clinical validation or lack of therapeutic implications. Finally, the statement discusses the pathological minimum requirements for tissue samples used in HRD testing, ensuring adequate sample quality and tumor content for reliable results.

**Conclusion:**

HRD testing is a valuable tool for personalizing ovarian cancer treatment, particularly in identifying patients who may benefit from PARPi therapy. However, assay selection, timing, and result interpretation require careful consideration. This statement provides a structured approach to optimize HRD testing, aiming to improve clinical decision-making and patient outcomes.

## Definition of HRD and HRD testing

Genomic instability (GIS) is one of the most common causes of tumorigenesis [1]. There are several DNA repair systems that play a significant role in maintaining genomic stability. If there is an imbalance or malfunction in these systems, often due to mutations, the genome exhibits instability. One of these DNA repair systems is the homologous recombination repair (HRR) system. When double strand breaks and interstrand cross-links (ICL) occur during genomic replication, the HRR system respond to these mutations with its proteins for repair.

Defects in HRR pathway due to (epi-) genetic events may result in the phenotype of homologous repair deficiency (HRD), indicating the inability to repair DNA double-strand breaks. If HRD occurs, GIS can be promoted. GIS may manifest as genomic loss of heterozygosity (gLOH), telomeric imbalance (TAI) and large-scale transitions (LST).

Especially in the tumorigenesis of high-grade serous ovarian cancer (HGSOC), the HRR system plays a significant role. Germline and somatic mutations within the breast-cancer gene *(BRCA) 1* and *BRCA 2* are mainly responsible for HRR pathway defects. Approximately 13 to 15% of patients with HGSOC show a germline mutation in *BRCA1/2*, and up to 3–7% show somatic mutations [2, 3]. However, besides *BRCA1* and *2*, there are other genes involved that may lead to HRD, such as *BRCA1*-associated RING domain 1 (*BARD1*), BRCA-interacting protein 1 (*BRIP1*), checkpoint kinase 1 (*CHEK1*), checkpoint kinase 2 (*CHEK2*), family with sequence similarity 175, member A (*FAM175A*), nibrin (*NBN*), partner and localizer of BRCA2 (*PALB2*), RAD51 paralog C (*RAD51C*), RAD51 paralog D (*RAD51D*), and many more.

The clinical impact of these malfunctions in the HRR pathway was demonstrated by the introduction of poly(adenosine diphosphate [ADP]–ribose) polymerase (PARP) inhibitors (PARPi). The PARPi block base excision repair, which leads to the accumulation of single-strand breaks during DNA replication. This ultimately results in a collapse of the repair system and the formation of double-strand breaks. In cells with HRD, these breaks cannot be adequately repaired, leading to synthetic letality in the presence of PARPi.

The efficacy of PARPi in maintenance therapy for HGSOC has been demonstrated in several studies, including those utilizing different drugs such as Olaparib monotherapy in the SOLO1 study, the combination of Olaparib and bevacizumab in the PAOLA1 study, and Niraparib monotherapy in the PRIMA trial, which led to EMA and FDA approval [4–7].

The *BRCA* germline mutations were the first to be understood as an indicator for the effective use of PARPi. The PAOLA1 trial showed that not only patients with pathogenic *BRCA1/2* mutations but also those with genomic instability measured by the Myriad MyChoice assay benefited from maintenance therapy with Olaparib. Therefore, the importance of other HRD-related genes is emphasized, and their inclusion in regular testing for patients with ovarian cancer (OC) is warranted. This would enable clinicians to make well-grounded clinical decisions regarding the use of PARPi. The aim of this statement is to simplify clinicians’ decision-making regarding indications and correct conduct of HRD testing in patients with OC based on current knowledge.

## Landscape of tests and its choice

There are different tests available for the determining of HRD status. Principally, there are three different categories for the determining HRD:

1. Next-generation sequencing (NGS) assays: These assays analyze genomic DNA to detect mutations in genes associated with HRD, such as *BRCA1* and *BRCA2*, as well as other HRD-related genes.
  - Genetic Testing: mutations in the *BRCA1* and *BRCA2* genes are well-established indicators of HRD, particularly in breast and ovarian cancers. Genetic testing can identify these mutations, and the presence of such mutations suggests HRD. This testing can be performed through various methods, including targeted sequencing, multiplex ligation dependent probe amplification (MLPA), or next generation sequencing (NGS).
  - Homologous Recombination Deficiency Score (HRD Score): some commercial tests, such as the Myriad myChoice^®^ HRD test, calculate an HRD score based on multiple genomic markers associated with HRD. This score is used to predict HRD and guide treatment decisions.This test includes measures of GIS such as loss of heterozygosity (LOH), telomeric allelic imbalance (TAI) and large-sclae state transitions (LSTs).
2. Genomic instability assays: these assays measure genomic instability through various methods, such as assessing loss of heterozygosity (LOH), telomeric allelic imbalance (TAI), and large-scale state transitions (LSTs).
  - Loss of Heterozygosity (LOH) Testing: LOH is a common feature of HRD and is characterized by the loss of one of the two copies of a gene in a tumor. LOH testing can identify regions of the genome where one copy has been lost, indicating HRD
  - Telomeric allelic imbalance (TAI) refers to an imbalance in the lengths of telomeres, which are the protective caps at the ends of chromosomes, between the two alleles of a gene. Telomeric allelic imbalance is a form of genomic instability that can be indicative of defects in DNA repair pathways, such as homologous recombination repair (HRR), and is associated with certain types of cancer, including ovarian cancer.
  - Large-scale state transitions (LSTs) are structural genomic alterations that occur on a large scale, involving changes in chromosomal structure or organization. These transitions may include events such as chromosomal rearrangements, copy number alterations, or changes in chromosome arm status.
3. Functional assays: these assays evaluate the functionality of HRD repair pathways in cells, often through laboratory-based experiments or assays measuring the ability of cells to repair DNA damage (e.g., RAD51 Foci assay).

The following tests are clinically approved and are commonly accessible in Germany:

**Table:** 

Test name	Mechanism	HRD-positive	Patient’s probe	Trial evaluated
Genetic testing				
*BRACAnalysis CDx test*	Germline mutation in *BRCA1/2*	Mutation detectable	Blood	
HRD-Test				
*Myriad myChoice CDx test*	Mutation status of *BRCA1/2* AND GIS (gLOH + TAI + LST)	*BRCA*-mutation OR GIS > 42	Tumor tissue	PRIMA/PAOLA-1/VELIA/NOVA
*FoundationOne CDx panel*	mutation status of *BRCA1/2* AND genomic instability (gLOH)	BRCA-mutation OR LOH-Score ≥ 16% [8]	Tumor tissue	ATHENA Mono/ARIEL 2/3/QUADRA
*NOGGO GIS assay* [9]	Next Generation Sequencing (NGS) hybrid-capture biomarker assay that detects *BRCA1/2* + 55 further HRR-relevant genes and structural alterations to establish GIS	BRCA-mutation or GIS > 83	Tumor tissue	PAOLA-1 cohort
*Geneva test* [10, 11]	OncoScan + number of large-scale state transitions (nLST)	nLST threshold of 15	Tumor tissue	PAOLA-1 cohort
*Academic Leuven HRD test* [12]	Targeted sequencing of genome-wide single-nucleotide polymorphisms and coding exons of eight HR genes including BRCA1, BRCA2, and TP53		Tumor tissue	PAOLA-1 cohort
SOPHiA DDM™ Dx HRD Solution [13]	Detects SNVs and Indels in 28 genes involved in the HRR pathway, including BRCA1 and BRCA2		Tumor tissue	PAOLA-1 cohort
Illumina TSO 500	Low-WGS for LOH + TAI + LST		Tumor tissue	PAOLA-1 cohort
BRCA-like classifier [14]	Discriminate BRCA-associated from sporadic cancers by employing the shrunken centroid algorithm; low-WGS	BRCA-like > 0.5; non-BRCA-like ≤ 0.5	Tumor tissue	PAOLA-1, AGO-TR1

Regarding the cutoff values of each test, we like to underline that the 95% CI are generaly not reported and the interpretation of values near the threshold should be discussed interdisciplary, considering various factors and clinical context.

Further tests that are not yet been clinically evaluated regarding progression-free survival (PFS) and overall survival (OS) but show a high concordance to the Myriad myChoice (reffered to as bridging) [15, 16] include:

- CytoSNP: Single Nucelotid Polymorphism (SNP) Array for LOH + TAI + LST.
- Affymetrix OncoScan: Single Nucelotid Polymorphism (SNP) Array for LOH + TAI + LST.
- OncoMine: Shallow Whole Genome Sequencing (low-WGS) for LOH-Score.
- AmoyDX: low-WGS HRD focus panel for the detection of BRCA1/2 mutation and GIS and many more.

Real-world data have shown that a genomic loss of heterozygosity (gLOH) (> 16%) und GIS (> 42) exhibit a significant overlap and are clinically comparable regarding the time to treatment discontinuation (TTD) [17].

## Statement

1. Since patients are considered HRD positive and thus eligible for maintenance therapy with olaparib either with a germline or somatic *BRCA1/2* mutation OR GIS positivity both GIS and *BRCA1/2* status has to be evaluated together for a conclusive result.
2. Since GIS is a continuous marker to which a hard cutoff is applied, ideally all assays used to stratify patients for treatment decisions should have shown effectiveness by providing clinical PFS and ideally OS data. However, in most settings, these data are not available due to limited access to clinical trial specimens.
3. A number of assays with their specific cutoffs have also been evaluated and validated with tissue samples of the PAOLA1 clinical trial, made available by the ENGOT/ARCAGY [18]. For some of these assays, PFS and OS data are available, and survival curves show highly comparable results with comparable Hazard ratios [9, 11, 18, 19]. Therefore, it is recommended to utilize assays for which a significant clinical benefit, and comparable PFS and OS advantages have been shown on samples from the PAOLA-1 trial or other clinical trials looking at outcome in HRD/HRP population.

## When is it appropriate to test for HRD in OC, and when is it not recommended?

All patients diagnosed with ovarian cancer should undergo genetic counseling and testing for *BRCA1/BRCA2* and other *BRCA*-related genes as recommended by guidelines. This testing is typically included in a broad standardized panel of the most common mutations associated with hereditary cancer syndromes.

The following criteria should be considered for further testing HRD in ovarian cancer:

- Contextual relevance: the inclusion of the HRD test must be made wihin the overall clinical context of the patient’s condition and treatment plan. The ability to take oral medications is mandatory since all PARP-inhibitors are used orally.
- Negative germline mutations: if germline mutations for *BRCA1/2* alone tested by human genetics are negative, HRD can be persued as second test in a two-step procedure. This can be performed immediately following a negative result for germline mutations. Ideally, HRD testing should be performed simultaneously with germline testing.
- Primary setting: HRD testing should ideally be conducted after the initial diagnosis using tumor tissue. If tumor tissue can not be obtained during cytoreductive surgery, multiple minimally invasive biopsies should be taken to gather sufficient material. The selection of biopsy anatomic sites should prioritize obtaining a high tumor sample size to optimize the results (further information below). Tissue can be collected via laparoscopy or interventional radiology.
- Histological subtypes: HRD testing is particularly relevant for histological subtypes such as high-grade serous, endometrioid, and clear cell epithelial ovarian carcinoma, as well as ovarian carcinosarcoma, following the WHO classification of 2014 (*p53*-mutated).

The following criteria are not eligible for further HRD testing:

- Positive germline mutation for *BRCA*: the use of additional HRD testing is obsolete.
- Recurrent disease or previously treated ovarian cancer: there is a lack in clinical and preclinical trials on this topic. HRD testing may not be reliable in the setting of recurrent disease or pretreated cancer, as alterations in tumor cells and the tumor microenvironment could affect the significance of results. Patients who have already received PARP inhibition or other immunogenic therapy during ovarian cancer treatment should be excluded from testing outside from clinical trials. However, therapies in other preexisting cancer sites are not part of this exclusion. If in recurrent disease a previous HRD test has been done, there is no need to reevaluate HRD again. If a HRD test has not been done before and if the result supports the treatment decision making process, it can be considered to be performed in a recurrence situation on an individual basis.Tumor tissue obtained after neoadjuvant chemotherapy within the primary diagnosis: the significance of the HRD test in tissue obtained after neoadjuvant chemotherapy at the time of primary diagnosis remains unclear. Tumor necrosis at the time of interval surgery can negatively influence the test results.
- Histological subtype: all forms of low-grade epithelial ovarian carcinoma following the WHO classification of 2014 are not eligible for further HRD testing.

## Minimum requirements and stardards for pathological examination

The molecular pathological report should include the following minimal information:

1. Patient identification and short clinical background:
  - Date of initial diagnosis.
  - Date of test performance.
  - Statement of previous administration of systematic treatment.
2. Details on the assay used:
  - Name of the assay.
  - Assay performance parameters (specification of mechanism used within the test).
  - Minimal and maximum tumor cell content.
  - Listing of all genes covered by test.
  - Sufficiency of sequencing depth (in case of NGS assays).
  - Cut-off and threshold for a test result.
  - Clear statement on clinical test approval, its eligibility for HRD testing and name of reference.
3. Details of the specimen taken for testing:
  - Histological diagnosis (confirmation that high-grade epithelial ovarian cancer is present).
  - Anatomic site of specimen taken.
  - Tumor cell content.
  - Statement on adequacy of sample measurements: an adequate sample should be at least 5 mm in diameter with at least 30% tumor cell count [20]. CAVE: in lymph node metastasis the tumor cell count can be low due to a high amount of immunocompentent cells (rapidly below 10%).
  - Cut-off definition: if the HRD score lies few points beneath the medical approval of a certain drug, a new biopsy to score a higher tumor cell count **can be** considered. Moreover, an off-label use of the drug **can be** discussed.
4. BRCA1/2 status:
  - Somatic mutation status of *BRCA1/2*, including large deletions and information on LOH of *BRCA1/2*
  - classification of *BRCA* mutation according to consensus recommendation of the American College of Medical Genetics and Genomics and the Association for Molecular Pathology
5. Genomic instability score as either positive or negative according to the test specific cut-off or threshold:
6. Final HRD status based on *BRCA1/2* mutation status, GIS and other HRR-relevant genes tested (HRD + vs HRD-).
7. If applicable, recommendation of specific drug and name of reference.

## Data Availability

No datasets were generated or analysed during the current study.
